# The association of psychosocial work quality with changes in the mental health of young adults starting career work

**DOI:** 10.5271/sjweh.4263

**Published:** 2026-03-01

**Authors:** Malte van Veen, Karen M Oude Hengel, Roosmarijn MC Schelvis, Cécile RL Boot, Karin Veldman, Iris Arends, Ute Bültmann

**Affiliations:** 1 Netherlands Organisation for Applied Scientific Research TNO, Unit Health and Work, Leiden, The Netherlands.; 2 Amsterdam UMC location Vrije Universiteit Amsterdam, Department of Public and Occupational Health, The Netherlands.; 3 Societal Participation and Health, Amsterdam Public Health research institute, Amsterdam, The Netherlands.; 4 Body@Work, Research Center on Work, Health and Technology, TNO/Amsterdam UMC, Amsterdam, The Netherlands.; 5 Amsterdam UMC location University of Amsterdam, Department of Public and Occupational Health, Amsterdam, The Netherlands.; 6 Department of Health Sciences, Community and Occupational Medicine, University Medical Center Groningen, University of Groningen, Groningen, The Netherlands.; 7 Arbo Unie, Knowledge Institute for Work and Health, Utrecht, The Netherlands.

**Keywords:** longitudinal fixed-effects regression, transition to work, young worker

## Abstract

**Objective:**

This study investigated whether (i) young adults’ mental health problems change when starting career work, (ii) potential changes in mental health problems differ by psychosocial work quality, and (iii) mental health problems during adolescence moderate potential changes in mental health by psychosocial work quality.

**Methods:**

We used data from the Tracking Adolescents’ Individual Lives Survey (TRAILS) cohort. Follow-up time was 2–4 years. Mental health was measured with the youth and adult self-report scales. Longitudinal fixed-effects regression analyses were applied to estimate within-person changes in mental health of young adults entering career work with good, moderate, or poor psychosocial work quality (N=850) and model adolescent mental health as effect modifier of this change (N=766).

**Results:**

When psychosocial job quality of the first career job was ignored, mental health problems did not significantly change among young adults after having entered career work compared with not having career work. Taking psychosocial job quality into account, mental health problems increased among young adults starting career work in poor psychosocial quality compared with not having career work (adjusted mean score increase 0.12, 95% confidence interval 0.03−0.21). No significant changes in mental health problems were found for young adults entering work with moderate-to-good psychosocial work quality. We found no evidence for adolescent mental health problems as moderator.

**Conclusion:**

Psychosocial work quality potentially plays a role for young workers’ mental health. Improving poor psychosocial work quality of young adults might contribute to a mentally healthier start of one’s working life.

Being in one’s 20s is a time in which the lives of most young adults change considerably. Despite young adults’ lives taking different paths during this time, most young adults eventually graduate and transition to working life (1, 2). In this study we are interested in what we call career work, as opposed to working in a side-job alongside education (1). The start of career work constitutes a transition in which work becomes central in the life of the young adult and marks the beginning of a new life phase (3). This transition can be accompanied by other substantial life changes like social and financial independence and leaving the parental or student home (4).

The transition into first career work might constitute a period of vulnerability in which young adults are particularly susceptible to exposures affecting their mental health (3, 5, 6). One potentially relevant exposure is the quality of the psychosocial work environment that young adults encounter in their first career job. For the general working population, previous studies showed that a work environment characterized by poor psychosocial work quality (eg, high job demands, low decision latitude, or violence) is associated with poorer mental health (7–10).

Previous research indicates that psychosocial work factors known to affect mental health of workers of all ages in general also play a role for young workers (11–14). At the same time, some aspects of the psychosocial work environment might be more impactful for younger workers’ mental health, ie, missing a sense of community at work (11) or experiencing a lack of autonomy (15, 16). Young workers are furthermore more likely to encounter unwanted conduct, including bullying and sexual harassment at work (17) However, to date, little is known about the role of psychosocial work quality concerning mental health when young adults start career work. Milner et al (18) showed that, in Australia, mental health improved for young adults who transitioned from not working into work with good psychosocial work quality while it deteriorated for those who transitioned into work with poor psychosocial work quality. These findings suggest that good psychosocial job quality might be a positive contributor to mental health in young adulthood. However, additional evidence based on different samples is necessary to draw more robust conclusions. Addressing this knowledge gap is practically relevant because poor mental health early in life can lead to lifelong disadvantages (19).

Additionally, a cross-sectional study showed that poor mental health during adolescence exacerbated the association of poor psychosocial work quality with mental health problems for young workers (20). Young adults who experienced poor mental health during adolescence and start career work in a job with poor psychosocial work quality might thus have an additional risk of experiencing mental health problems. The role of adolescent mental health however has not yet been studied in the context of transitioning into career work. By analyzing adolescent mental health problems as a potential effect modifier, we might be able to better understand which young adults are more or less affected by good or poor psychosocial work quality when first starting career work. This knowledge can help prevent the aggravation of mental health problems and related consequences, such as impaired work ability and productivity losses (8) early in the life course.

The aims of the current study were to investigate by means of self-report whether (i) young adults’ mental health problems change when starting career work, (ii) potential changes in mental health problems differ by psychosocial work quality, and (iii) mental health problems during adolescence moderate potential changes in mental health problems by psychosocial work quality for young adults starting career work.

## Methods

### Data and study sample

Data were used from the TRracking Adolescents’ Individual Lives Survey (TRAILS study), an ongoing prospective population-based cohort study currently consisting of seven waves. In 2000, individuals born 1989–1991 and living in three northern provinces in The Netherlands were invited to TRAILS; 2229 individuals participated in the cohort. For the initial wave, participants’ parents were approached via a telephone call after the participants’ school decided to participate in the recruitment process. TRAILS contains data on participants’ psychological, social and physical development. The participants’ average age of the last included wave was 29 years. The initial response rate of the TRAILS study was 76% and the retention rates in the fifth, sixth and seventh waves were 80%, 73%, and 55% of the baseline sample, respectively. All variables that are used in the current study are based on self-reports. More detailed information about the TRAILS study is published elsewhere (21).

Two observations per young adult – before and after the transition to career work – were included to address research aims 1 and 2. The period between two waves was 2–4 years. We only included participants who started career work in either wave five, six, or seven (average age at measurement 22, 26, and 29 years) because psychosocial work quality was measured in these three waves. Career work was defined as working ≥12 hours per week and not being in education, both measured with self-reports. For 212 individuals, it was not clear in which wave they transitioned into first career work due to missing data on work status. By using the event history calendar data for work history (measured in wave seven), we determined the wave of transition to career work for 111 of these individuals. The remaining 101 individuals with no information on start of career work were excluded. Next, we excluded individuals with missing data on psychosocial work quality (N=75). Moreover, individuals with missing data on mental health problems at either measurement were excluded (N=70). Finally, we excluded those with missing data on time-varying confounders at either measurement wave (N=32), resulting in a final sample of 850 participants with complete data to answer the first and second research question.

**Figure 1:**
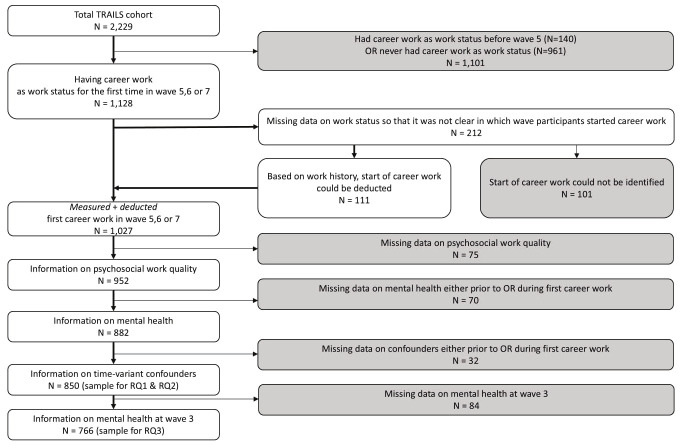
Flow chart of the selection of the study population.

To investigate the role of mental health during adolescence as effect modifier (research aim 3), we additionally included each participant’s observation from wave three (average age at measurement 16 years). This information was missing for 84 individuals, leading to a sample size of 766 participants for the research aim 3 (figure 1). See supplementary material, URL, figure S1 for a timeline of the measurements and table S1 for a comparison of the sample with individuals excluded based on missing data.

### Mental health problems

Mental health problems were assessed using the internalizing problems scale from the Adult Self-Report (ASR) (ie, anxious/depressed, withdrawn/depressed and somatic problems) (22). For this scale, participants reported internalizing problems over the last six months via 39 items (eg, “I feel alone" or “Other people don’t like me") on a three-point Likert scale (0 = not true, 1 = somewhat or sometimes true, 2 = very true or often true). The ASR score for internalizing problems is the average response to all items. The total scale ranges from 0 (no problem ever applies) to 2 (all problems apply “very often"), with higher scores indicating more mental health problems.

### Psychosocial work quality

Psychosocial work quality was retrieved from the wave in which young workers had their first career work. In the TRAILS study, the psychosocial work environment is measured by five factors from the short version of the Copenhagen Psychosocial Questionnaire (COPSOQ II) (23), namely decision authority, development opportunities, meaning of work, work pace, and quantitative demands. In the current study, these five factors were used for a comprehensive assessment of psychosocial work quality. All items for all factors were coded in a way that higher scores reflected lower psychosocial work quality. Participants had to indicate to what extent statements concerning the five psychosocial work factors apply to them, ranging from 0 (“very low extent") to 4 (“very high extent"). All factors were measured with two items except for decision authority and development opportunities in wave 7, for which the measurement was done with one item each. In line with Milner et al (18), we operationalized psychosocial work quality as the number of psychosocial job adversities. For the current study, each psychosocial work factor was firstly dichotomized into no adversity (“very low extent", “low extent", “somewhat": coded 0) or adversity (“high extent", “very high extent": coded 1). The sum score of these dichotomized indicators formed an ordinal composite scale of psychosocial work quality, range 0–5. Because of the low number of participants with three (N=19), four (N=4), or five psychosocial job adversities (N=0), these groups were combined into one group. This resulted in three levels of 0, 1 or ≥2 psychosocial job adversities, which we labelled as good, moderate, and poor psychosocial work quality, respectively.

### Adolescent mental health problems

To analyzing the moderating effect of adolescent mental health problems, internalizing problem scores were retrieved from wave three. This was measured with the internalizing problem scale of the Youth Self-Report (YSR), which is the youth version of the ASR (24).

### Time-variant confounders

Age, physical health, and marital status were included as potential time-variant confounders. Age was included as continuous variable (in years) because the incidence of mental health problems has been shown to increase up to the age of 30 (25) and young adults who are older when they start career tend to have an occupation characterized by a better psychosocial work environment (26). Physical health was included because poor physical health is likely to lead to both poorer mental health and a poorer psychosocial work environment via selection effects (20). Physical health was measured with the single item “how do you rate your physical health during the last 30 days?". Answer options differed across the waves and were harmonized to poor, moderate, and good physical health. Marital status was included because having a partner has been shown to be beneficial to mental health at least in the short run (27), and it is plausible that having a partner relates to having more financial resources increasing the likelihood of being able to choose a job with a more favorable psychosocial work environment. Marital status is a dichotomous variable with response options “single" and “in a relationship".

### Statistical analyses

Percentages and means with standard deviations (SD) were used to describe demographic characteristics (sex and educational attainment), time-variant confounders, psychosocial work quality and mental health problems. Educational attainment was categorized into low (primary, lower vocational and lower secondary education), medium (intermediate vocational and intermediate secondary education) and high (higher secondary, higher vocational education and university).

Linear fixed effects regression analyses (28) were applied to model within-person changes in mental health problems of the participants. First, the changes in mental health were modelled regardless of psychosocial work quality, hereafter changes in mental health grouped by good, moderate, and poor psychosocial work quality were analyzed. Fixed effects models control for any time-invariant characteristics of a young adult (eg, sex, cognitive capacity, general tendency to experience unpleasant emotions, early childhood experiences). As such each individual acts as their own control (see equation A for the analysis including groups of different psychosocial work quality, see the supplementary equation for the first analysis disregarding work quality).


Equation A^1^: $${\mathrm{Mental}\mathrm{Health}}_{\text{it}}\text{= }{\beta 1\text{*}\mathrm{Psychosocial}\mathrm{Work}\mathrm{Quality}}_{\text{it}}\text{+}{\beta 2\text{*}X}_{\text{it}}\text{+}\mu_{i}\text{+}\varepsilon_{\text{it}}$$

^1^ Mental Health_it_ is the individual mental health score predicted by psychosocial work quality (poor, moderate, or good, compared with not having started career work) (Psychosocial Work Quality_it_), a person-specific error term μ_i_ controlling for all time-invariant variation (using de-meaning), time-variant confounders X_it_ and a residual error term ε_it_.

Equation A only informs about within-group changes without comparing the change in mental health problems between groups. For testing whether the change in mental health problems differed significantly between psychosocial work quality subgroups, we fitted an additional fixed effects regression model with an interaction term defined as the product of a work status indicator and a *time-invariant* psychosocial work quality indicator (equation B). The interaction term could not just be added to equation A because this would result in multicollinearity as prior to career work *every* participant would have the value “not having started career work" for *Psychosocial Work Quality as defined in equation A* and “not at work" for *Work Status Indicator*.


Equation B^1^: $$\begin{array}{c} {\mathrm{Mental}\mathrm{Health}}_{\text{it}}\text{=}{\beta 1\text{*}\mathrm{Psychosocial}\mathrm{Work}\mathrm{Quality}\mathrm{Indicator}}_{i}\text{*}\\ {\mathrm{Work}\mathrm{Status}\mathrm{Indicator}}_{t}\text{ +}{\beta 2\text{*}X}_{\text{it}}\text{+}\mu_{i}\text{+}\varepsilon_{\text{it}} \end{array}$$

^1^ Mental Health_it_ is the individual mental health score predicted by the product of a time-invariant indicator for the psychosocial work quality of the first career job (poor, moderate, or good) (Psychosocial Work Quality Indicator_i_) and work status (at work, not at work) (Work Status Indicator_t_), a person specific error term μ_i_ controlling for all time-invariant variation (using de-meaning), time-variant confounders X_it_ and a residual error term ε_it_.

For modelling the moderating effect of adolescent mental health problems on change in mental health problems due to psychosocial work quality, equation A is expanded with an interaction term, which is the product of adolescent mental health problems and psychosocial work quality (equation C). By adding this term, we assessed whether the change in mental health when starting career work with different psychosocial job quality depends on mental health during adolescence.


Equation C^1^: MentalHealthit= β1*PsychosocialWorkQualityit+β2*PsychosocialWorkQualityit* AdolescentMental Health i+β3*Xit +μi+εit

^1^ Equation A expanded by interaction term; The main effect of adolescent mental health is not included. This is because this factor is, by definition, time-invariant and thus receives a coefficient of 0 when estimated using fixed-effects.

We decided to conduct additional between-group analyses to provide context to our focal results after having obtained results from the fixed-effects regression analyses. We plotted the average mental health scores from the waves preceding and succeeding the transition to career work respectively, grouped by psychosocial work quality based on estimated marginal means from the adjusted model. We also tested the between-group differences for each work status using two separate ANOVA’s with Tukey’s HSD post-hoc tests.

## Results

### Description of participants

Participants were predominantly female (60.8%), and more than half (56.9%) had high educational attainment when they first started career work (table 1). The average age in the wave preceding first career work was 21.2 (SD 2.3) years and it was 24.6 (SD 2.4) years in the wave in which participants had first started career work. After the transition to career work, 55.3% of the young workers reported good psychosocial work quality, 32.6% reported moderate psychosocial work quality, and 12.1% reported poor psychosocial work quality.

**Table 1:** Descriptive table of sociodemographic variables, internalizing problems, and time-variant confounders by work status (N=850). [NA=not applicable; SD=standard deviation].

		Prior to first career work			After transition to first career work	
Characteristic		% (N)	Mean (SD)		% (N)	Mean (SD)
Sex female ^a^		60.8 (517)			60.8 (517)	
Educational attainment after transition to first career work ^a^						
	Low				4.8 (41)	
	Medium				37.8 (321)	
	High				56.9 (484)	
	Missing				0.5 (4)	
Age in years			21.2 (2.3)			24.6 (2.4
Physical health						
	Good	88.5 (752)			86.0 (731)	
	Moderate	9.5 (81)			12.2 (104)	
	Poor	2.0 (17)			1.8 (15)	
Marital status						
	In relationship	40.7 (346)			61.1 (519)	
	Single	59.3 (504)			38.8 (330)	
	Missing	0			0.1 (1)	
Psychosocial work quality						
	Good (0 adversities)	NA			55.3 (470)	
	Moderate (1 adversity)	NA			32.6 (277)	
	Poor (≥2 adversities)	NA			12.1 (103)	
Internalizing problems						
	Whole sample		0.25 (0.24)			0.28 (0.24)
Internalizing problems (psychosocial work quality)						
	Good (0 adversities)		0.24 (0.2)			0.25 (0.2)
	Moderate (1 adversity)		0.26 (0.2)			0.29 (0.3)
	Poor (2+ adversities)		0.28 (0.2)			0.38 (0.3)
	Whole sample at wave 3 ^b^		0.32 (0.24)			NA

^a^ Sex (female versus male) and educational attainment are based on self-reports and are not included in fixed-effects regression models. ^b^ Age at wave 3: 16 years, N=766.

### Change in mental health problems when transitioning to career work

The increase in internalizing problems scores among young adults starting career work was not statistically significant after controlling for confounders (0.06; 95% CI -0.02−0.15; table 2).

**Table 2:** Changes in internalizing problems of 850 young adults transitioning into first career work based on linear fixed effects regression analysis; reference status: “not having career work”. **Statistically significant change in bold**. Results presented for each psychosocial work quality subgroup are based on equation A from the methods section. [CI=confidence interval]

		Crude model				Adjusted model ^a^		
		N	Average within-person change	95% CI		N	Average within-person change	95% CI
Whole sample		**850**	**0.02**	**0.01−0.04**		850	0.06	-0.02−0.15
Per psychosocial work quality								
	Good (0 adversities)	470	0.01	-0.01−0.02		470	0.04	-0.05−0.13
	Moderate (1 adversity)	**277**	**0.03**	**0.01−0.06**		277	0.07	-0.02−0.16
	Poor (≥2 adversities)	**103**	**0.09**	**0.05−0.13**		**103**	**0.12**	**0.03−0.21**

^a^ Included time-variant confounders were age, physical health, and marital status.

For young adults transitioning into career work with poor or moderate psychosocial work quality, internalizing problems increased significantly in the crude model, while no statistically significant change was found for the group of young adults transitioning to career work with good psychosocial work quality (table 2). The adjusted model showed the same pattern as the crude model, but internalizing problems only increased significantly for young adults who transitioned into career work with poor psychosocial quality (0.12; 95% CI 0.03−0.21; table 2).

The increase in internalizing problems (ie, the difference in slopes) was significantly larger for young adults starting career work in poor compared with moderate-to-good psychosocial work quality (table 2).

Additional between-person analyses (see supplementary figure S2) showed that internalizing problem scores before starting career work did not significantly differ between groups entering career work with poor or moderate psychosocial work quality (supplementary table S2). After having started career work, young workers who did so in poor psychosocial work quality had significantly higher internalizing problems scores compared with those in moderate-to-good psychosocial work quality (both P<0.01; supplementary table S2), while no differences were found between the groups with good versus moderate psychosocial work quality (P=0.08; supplementary table S2).

**Table 3:** Between-group differences of within-person changes of internalizing problem scores (ie, differences in slopes). **Statistically significant differences in bold**. Results presented here are based on equation B from the methods section. [CI=confidence interval]

Comparison of within-person change between subgroups			Crude model				Adjusted model ^a^		
			Interaction coefficient	95% CI	P-value		Interaction coefficient	95% CI	P-value
Good psychosocial work quality (reference)									
	vs	Moderate psychosocial work quality	0.03	-0.01−0.06	0.09		0.03	-0.01−0.06	0.08
	vs	Poor psychosocial work quality	**0.09**	**0.04−0.13**	**<0.01**		**0.08**	**0.04−0.13**	**<0.01**
Moderate psychosocial work quality (reference)									
	vs	Poor psychosocial work quality	**0.06**	**0.01−0.11**	**0.01**		**0.05**	**0.01−0.10**	**0.03**

^a^ Included time-variant confounders were age, physical health, and marital status.

### Adolescent mental health problems as effect modifier

In the crude model, the increase in internalizing problems was higher for young adults starting career work with moderate psychosocial work quality when they experienced more internalizing problems during adolescence (0.12 95% CI 0.01−0.23; table 4), but it was no longer significant in the adjusted model (0.11 95% CI -0.01−0.22). No interaction effects in the crude or adjusted models were found for workers entering career work with poor or good psychosocial work quality.

**Table 4:** Effect moderation by mental health problems prior to starting first career work by psychosocial work quality group (crude and adjusted model) based on linear fixed effects regression analysis. Statistically significant change in bold (N=766). Results presented here are based on equation C from the methods section. [95% CI=95% confidence interval]

	N	Crude Model			Adjusted model ^a^	
		Coefficient for effect moderation	95% CI		Coefficient for effect moderation	95% CI
Good psychosocial work quality × Adolescent mental health problems	434	-0.03	-0.11−0.05		-0.04	-0.13−0.04
Moderate psychosocial work quality × Adolescent mental health problems	**240**	**0.12**	**0.01−0.23**		0.11	-0.01−0.22
Poor psychosocial work quality × Adolescent mental health problems	92	-0.09	-0.26−0.07		-0.11	-0.27−0.05

^a^ Included time-variant confounders were age, physical health, and marital status.

See supplementary table S3 for a sensitivity analysis, repeating the results displayed in table 2 based on the 766 individuals for which information on mental health prior to starting first career work was available.

## Discussion

Our study showed that young adults in general did not experience a change in mental health problems when starting career work compared with not being at work. When differentiating by psychosocial work quality, however, mental health problems increased for those in jobs with poor psychosocial work quality. Mental health problems during adolescence did not moderate the effect of psychosocial work quality on changes in mental health when starting career work.

In line with Milner et al (18), our study showed that mental health problems increase for young workers who experience poor psychosocial work quality at the beginning of their careers and suggested that the increase of mental health problems among young workers is greater when the psychosocial work quality is poorer, albeit that the increases we observed were of a very small magnitude. In contrast to the results by Milner et al (18), we did not find that mental health problems decreased when starting career work in good psychosocial working conditions. A possible explanation for this difference may be that our study concentrated on the short-term effects of entering career work while Milner et al (18) included more waves of data after the transition to career work. Future research could aim at disentangling possible differences between the short-lived versus more permanent changes in mental health and the role of the psychosocial work quality therein.

When looking at young workers transitioning to career work with poor psychosocial work quality, the mental health problems increased slightly (with 0.12 points on the ASR scale). Although the increase was rather small, this deserves attention. For the ASR scale, a so-called 'borderline range' is described in the literature, which is set at a minimum value of 0.52. An individual scoring this high is advised to receive further assessment for mental health problems (29). In the present study, none of the psychosocial work quality groups reached the borderline score shortly after transitioning to career work. However, those who start career work in poor psychosocial work quality might enter the borderline range at the next measurement occasion if the observed increase in mental health problems continues at the same rate. It can be speculated that, particularly among young workers remaining in jobs with poor psychosocial job quality, the increase of mental health problems might persist. Nevertheless, more research is needed as mental health problems might also attenuate once young workers get used to their psychosocial work environment.

Our additional between-person analyses comparing young workers based on the psychosocial work quality of their first career jobs suggest that the work environment might play a role in widening the differences in mental health problems between groups of workers over time. These analyses further carefully hint at selection effects in the sense that a young adult's mental health problems prior to starting their career might increase the likelihood that they end up in work with poor psychosocial work quality (29). The rank order of the psychosocial work quality groups concerning internalizing problems is already observable before the transition to career work. Those young adults starting and continuing to work in jobs with poor psychosocial work quality might thus find themselves in a downward spiral of increasing mental health problems (30).

In contrast with LaMontagne et al (20), we did not find that mental health during adolescence moderates the impact of psychosocial work quality on the mental health of young workers entering paid employment. As research is still scarce in this field, this calls for further research adopting a life course approach in which an individual’s mental health history and non-work related life-events are taken into account already before the transition to career work. A life course approach may help to better understand young adults’ situation at the start of their career and how psychosocial work quality affects mental health (5, 14, 31).

Our study contributes to a better understanding of mental health changes at the beginning of career work life as it is one of the few to date analyzing within-person effects and the role of adolescent mental health problems. A strength of our study is its use of a composite score to operationalize the psychosocial work exposure because research has shown that no one "toxic psychosocial work factor" exists that by itself determines mental health problems (9). By using a composite score, we can account for that different factors might determine the association of psychosocial work quality with mental health problems for different young workers. However, we need to acknowledge that the operationalization of psychosocial work quality had some limitations in the current study. The included psychosocial work factors are relevant for young workers as previous research suggests that psychosocial work factors known to be relevant to the general working population also affect young workers (11–16). Nevertheless, not all aspects of the psychosocial work environment that have been suggested to be particularly impactful for young workers [eg, sense of community at work, being responsible for others (11), or lack of autonomy (16)] or that young workers are more exposed than older workers [eg, to unwanted sexual advances at work (17)] were measured in the TRAILS study and could there not be incorporated in our composite score. However, previous research suggested a general pattern that good and poor psychosocial work factors respectively are tightly clustered (32), which has also been observed for young workers, albeit to a lesser extent (33). Assuming such clustering, not incorporating a potentially influential factor is unlikely to have biased our results. Additionally, composite scores were shown to be informative in previous studies which observed an association between psychosocial job quality and mental health problems based on composite scores (34–36). The study's second strength is the focus on within-person effects by using fixed effects regression analysis, which controls for all potential time-invariant confounders. The distinction of within- and between person effects is important because the two effects are not necessarily congruent (37, 38). A previous study showed, for example, that higher job autonomy was associated with lower emotional exhaustion between workers, but an increase in job autonomy led to an increase in emotional exhaustion within workers (39). Despite having addressed those confounders that we considered to be most relevant, we cannot rule out the risk of residual confounding due to time-variant variables that were not included in our adjusted models.

A limitation of our study is that we cannot rule out that our results are subjected to common method bias and potential reverse causality as both mental health problems during first career work and psychosocial work quality were measured during the same wave. Measures of psychosocial work quality might be affected by contemporaneous mental health problems. This is also labelled the gloomy perception effect, which implies that a young worker with poor mental health will perceive and report the psychosocial work environment as more negative than someone with better mental health even when they work in the same psychosocial work environment (30). In general, it is however assumed that subjective measures of the psychosocial work environment are not merely individual appraisals but reflect objective features of the psychosocial work environment (20). A final limitation is that the TRAILS study is subject to attrition bias. Male participants and participants whose parents had lower educational level are less likely to participate over time (2). Our sample comprised only few young workers with low educational attainment. Furthermore, compared to participants starting career work with moderate-to-good psychosocial work quality, relatively few participants were in the poor psychosocial work quality group. We have no reason to expect that this attrition led to a systematic bias of our results, but it potentially limits generalizability of our findings concerning these groups.

In conclusion, our study provides suggestive evidence that mental health problems increase among young workers who experience poor psychosocial work quality in their first career work. Improving poor psychosocial work quality of young adults might eventually contribute to a mentally healthier start of their working life.

## Supplementary material

Supplementary material

## Data Availability

Data may be obtained from a third party and are not publicly available. TRAILS data of the T1, T2, T3, T4 and T5 measurement waves are deposited in the Data Archiving and Networked Services of the Royal Dutch Academy of Sciences (DANS-KNAW) and access can be requested at www.dans.knaw.nl.
