# Supplementary material for: The association of psychosocial work quality with changes in the mental health of young adults starting career work
Source: Scand J Work Environ Health. 2026 Feb 28;52(2):169–78. doi: 10.5271/sjweh.4263 (PMC12962325; doi:10.5271/sjweh.4263)
Supplement: Supplementary material [file SJWEH-52-169-S001.pdf]

The association of psychosocial work quality with changes in the mental health of young adults starting career work[1]

by Malte van Veen, MSc,[2] Karen M Oude Hengel, PhD, Roosmarijn MC Schelvis, PhD, Cécile RL Boot, PhD, Karin Veldman, PhD, Iris Arends, PhD, Ute Bültmann, PhD

1. Supplementary material
2. Correspondence to: Malte van Veen, TNO, Unit Health and Work, Postbus 3005, 2301 DA Leiden, The Netherlands. [E-mail: malte.vanveen@tno.nl]

Figure S1 – Timeline with variables at different measurement moments

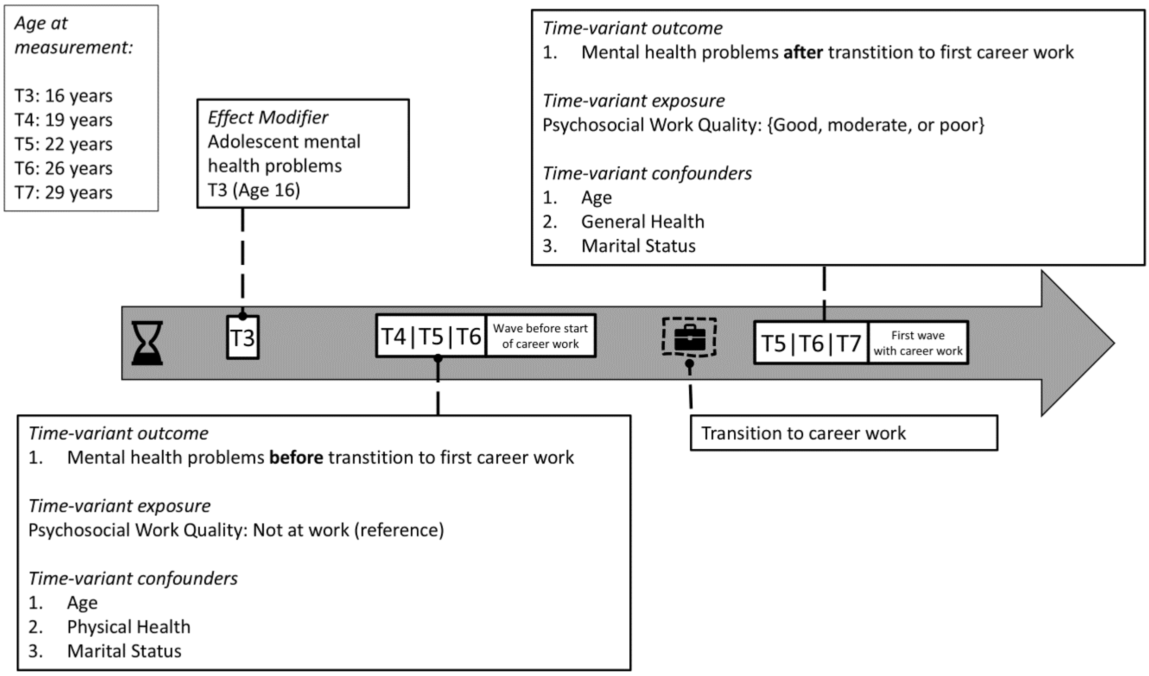

Figure S2 – Between group differences before and after having started first career work. Marginalized means of mental health problems before and during first career work grouped by psychosocial work quality, controlling for time-variant confounders age, physical health, and marital status (n=850).

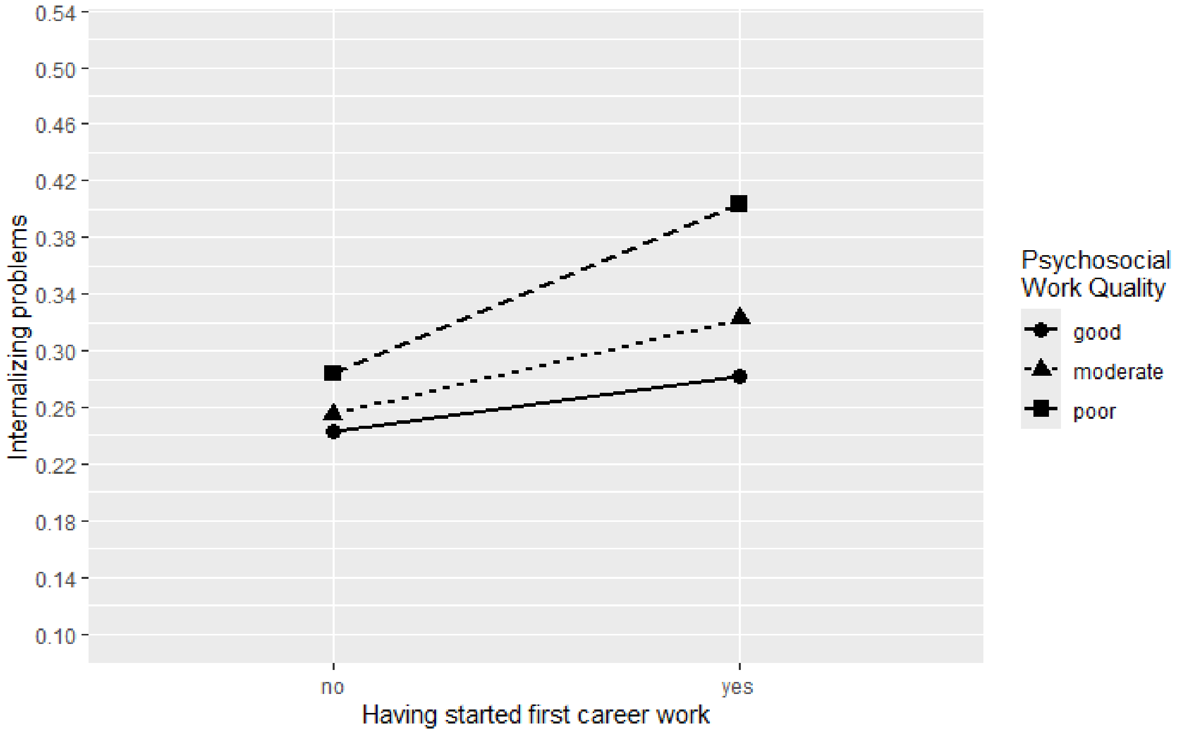

Table S1 Comparison of the sample with individuals excluded based on missing data

The table below shows the descriptives of the 278 individuals that were excluded due to missing data, but for whom we could determine in which wave they started with career work. Based on this information, it becomes apparent that prior to first career work compared to our study sample (information provided in table 1), individuals with missing data were more likely to be male χ^2 (1, N=925) = 10.76, p = 0.001, on average had a lower education χ^2 (2, N=1049) = 87.11, p < 0.001, and on average had higher internalizing problems (t 1126 = 4.13, p < .001). Age, general health and marital status can be considered to be comparable between the groups, all p's >0.05.

	Prior to first career work		After transition to first carreer work	
	(N=278)	valid	(N=278)	valid
**Sex**				
Female	138 (49.6%)		138 (49.6%)	
Male	140 (50.4%)		140 (50.4%)	
**Education**				
Low	46 (16.5%)	22.7%	45 (16.2%)	16.3%
Medium	32 (11.5%)	15.8%	107 (38.5%)	38.8%
High	125 (45.0%)	61.6%	124 (44.6%)	44.9%
Missing	75 (27.0%)	203	2 (0.7%)	276
**Internalizing Problems**				
Mean (SD)	0.323 (0.299)		0.291 (0.296)	
Missing	110 (39.6%)		41 (14.7%)	
**Age**				
Mean (SD)	21.7 (2.86)		25.2 (2.89)	
Missing	37 (13.3%)		0 (0%)	
**Physical Health**				
Good	139 (50.0%)	88.0%	185 (66.5%)	83.3%
Moderate	16 (5.8%)	10.1%	30 (10.8%)	13.5%
Poor	3 (1.1%)	1.9%	7 (2.5%)	3.2%
Missing	120 (43.2%)	158	56 (20.1%)	222
**Marital Status**				
In relationship	66 (23.7%)	38.6%	170 (61.2%)	63.4%
Single	105 (37.8%)	61.4%	98 (35.3%)	36.6%
Missing	107 (38.5%)	171	10 (3.6%)	268

Supplementary table S2. Between group differences in internalizing problem scores before and after the transition to first career work. Statistically significant differences in bold.

**Before starting first career work**				
Subgroup comparison		Mean Diff.	95% CI	p-value
Good psychosocial work quality (reference)				
vs	Moderate psychosocial work quality	0.01	-0.03−0.05	0.78
vs	Poor psychosocial work quality	0.04	-0.02−0.10	0.25
Moderate psychosocial work quality (reference)				
vs	Poor psychosocial work quality	0.03	-0.04−0.09	0.54
**After transition to first career work**				
Subgroup comparison		Mean Diff.	95% CI	
Good psychosocial work quality (reference)				
vs	Moderate psychosocial work quality	0.04	-0.01−0.08	0.08
**vs**	**Poor psychosocial work quality**	**0.12**	**0.07−0.19**	**<0.01**
Moderate psychosocial work quality (reference)				
**vs**	**Poor**	**0.09**	**0.02−0.15**	**<0.01**

Supplementary table S3. Changes in mental health problems of 766 young adults transitioning into first career work by psychosocial work quality group (crude and adjusted model) based on linear fixed effects regression analysis. Statistically significant change in bold. [95% CI=95% confidence interval]

	**Crude Model**			**Adjusted model[a]**		
Psychosocial Work Quality	n	Average within-person change in mental health problems	95% CI	n	Average within-person change in mental health problems	95% CI
Good (0 adversities)	434	0.01	-0.01−0.03	434	0.04	-0.06−0.13
Moderate (1 adversity)	**240**	**0.03**	**0.00−0.06**	240	0.06	-0.04−0.15
Poor (2+ adversities)	**92**	**0.08**	**0.03−0.12**	**92**	**0.10**	**0.00−0.20**

[a]Included time-variant confounders were age, physical health, and marital status

Supplementary Equation for first analysis disregard psychosocial work quality

$$Mental\ Health_{it} = \beta 1 * Work\ Status\ Indicator_{it} + \beta 2 * X_{it} + \mu_i + \varepsilon_{it}$$

[1]Mental $Health_{it}$ is the individual mental health score predicted by work status indicator (at work vs not at work), a person specific error term μ_i controlling for all time-invariant variation (using de-meaning), time-variant confounders X_{it} and a residual error term ε_{it}.
